# APOE Gene Associated with Dementia-Related Traits, Depression, and Anxiety in the Hispanic Population

**DOI:** 10.3390/genes14071405

**Published:** 2023-07-06

**Authors:** Chun Xu, Victoria Padilla, Stephanie Lozano, Daniela Gamez, Brenda Bin Su, Xuan Wang, Gladys Maestre, Kesheng Wang

**Affiliations:** 1 Department of Health and Biomedical Science, College of Health Affairs, University of Texas Rio Grande Valley, Edinburg, TX 78539, USA; victoria.padilla01@utrgv.edu (V.P.); daniela.gamez01@utrgv.edu (D.G.); 2 Department of Science, Graduate College of Biochemistry and Molecular Biology, University of Texas Rio Grande Valley, Edinburg, TX 78539, USA; stephanie.lozano01@utrgv.edu; 3 Department of Pediatrics, Division of Immunology, Allergy, and Retrovirology, Allergy and Immunology Baylor College of Medicine, William T. Shearer Center for Human Immunobiology, Texas Children Hospital, Houston, TX 77030, USA; bin.su@bcm.edu; 4 Department of Information Systems, Robert C. Vackar College of Business and Entrepreneurship, University of Texas Rio Grande Valley, Edinburg, TX 78539, USA; xuan.wang@utrgv.edu; 5 Neuroscience and School of Medicine, University of Texas Rio Grande Valley, Edinburg, TX 78539, USA; gladys.maestre@utrgv.edu; 6 Department of Family and Community Health, School of Nursing, Health Sciences Center, West Virginia University, Morgantown, WV 26506, USA

**Keywords:** dementia, neuropsychiatric disorders, APOE gene, Hispanic population, aging, demographic factors

## Abstract

Alzheimer’s disease (AD), a main cause of dementia, is commonly seen in aging populations with a strong genetic component. AD is one of the most common neurodegenerative disorders; it is a genetically and clinically heterogeneous disease. Specific demographic factors and genetic variants have been identified in non-Hispanic populations; however, limited studies have observed the Hispanic population. Therefore, we focused on investigating a known gene, APOE, associated with AD-related phenotypes and two psychiatric diseases (depression and anxiety) within the U.S. Hispanic population in our current study. A total of 1382 subjects were studied based on data collected from the Texas Alzheimer’s Research and Care Consortium (TARCC, *N* = 1320) and the Initial Study of Longevity and Dementia from the Rio Grande Valley (ISLD-RGV, *N* = 62). Questionnaires regarding demographics, medical history, and blood/saliva samples were collected. We genotyped the APOE gene. The current findings indicated that APOE-ε4 was associated with not only AD (*p* < 0.0001) but also with anxiety (*p* < 0.0001) and depression (*p* = 0.0004). However, APOE-ε3 was associated with depression (*p* = 0.002) in the Hispanic population. We provide additional evidence in which APOE-ε4 increased the risk for AD in Hispanics. For the first time, APOE alleles show increased risks for anxiety and depression in Hispanics. Further research is warranted to confirm the current findings.

## 1. Introduction

Alzheimer’s disease (AD) is one of the most common dementias. In 2015, 46.5 million people were diagnosed with dementia [1]. It is projected that every 20 years, the cases will approximately double, with an estimated 74.7 million cases by 2030 to 131.5 million cases by 2050 [1]. Behaviors and chronic diseases that increase the risk for dementia include smoking, a poor diet, a sedentary lifestyle, hypertension, diabetes, hypercholesterolemia, high BMI, depression, anxiety, and low cognitive engagement [2,3]. AD is a progressive disorder that disrupts the ability to carry out simple daily tasks and normal cognitive thinking. It may cause neuropsychiatric disorders including depression, anxiety, and apathy [4,5]. The cause of AD is not well understood.

Mild cognitive impairment (MCI) is an intermediate point between normal cognitive aging and untypical cognitive aging. MCI may interfere with daily life activities and cause symptoms such as forgetting details (e.g., dates, names, or events), losing belongings, and confusion, amongst other memory-related issues, but overall, a person with MCI can carry out normal daily tasks [6]. The typical age of onset for patients with MCI is 65+, and it affects approximately 10–20% of adults in the U.S. [7,8]. Hispanic adults who develop dementia-related traits tend to be younger (around the age of 45) compared to those belonging to other ethnic groups [9]. In addition, MCI is a risk factor for dementia and is more common in males than in females [10,11]. According to a study, 9.8% of Hispanics are affected with MCI [12]. Data from the CDC predict that U.S. Hispanics will experience a significant increase in dementia-related conditions regarding the population’s growth. Furthermore, U.S. Hispanics have demonstrated higher rates of memory disturbance and cognitive decline than non-Hispanic Whites [13,14].

AD is often comorbid with neuropsychiatric conditions. Some neuropsychiatric disorders are considered early indicators of AD and present when there are cognitive problems (e.g., MCI) that may lead to AD [15]. Moreover, age and AD progression can increase the susceptibility to neuropsychiatric symptoms [16]. We have recently reported that dementia is associated with several neuropsychiatric symptoms in a Hispanic cohort from the Maracaibo Aging Study in Venezuela [17]. Among the Hispanic population, the prevalence of psychiatric disorders is slightly higher in females (30.2%) compared to males [18]. Hispanics born in the U.S. were at a higher risk of neuropsychiatric disorders than Hispanics who were not U.S.-born [18].

Neuropsychiatric disorders are reported to be frequent in the Hispanic population, including anxiety, which is a disruptive feeling of dread, fear, and uncertainty with the anticipation of a future threat [19] and is the most common psychiatric disorder in the U.S. [20]. It has been estimated that 33.7% of the general population have been affected by anxiety in their lifetime [21]. Whites were the most commonly diagnosed group with anxiety when compared to Hispanics [22,23]. Another commonly seen psychiatric disorder is depression, which originates from the interactions of genes and environmental events, such as stressful/negative events [24]. Depression in late life could be associated with cognitive impairment and/or increased risk for dementia-related diseases [25]. It has been estimated that 16.6% of adults experience depression in their lives [26]. A meta-analysis study composed of 48 studies in America, Europe, and Asia showed that depression was the second leading neuropsychiatric disorder among patients with AD who had comorbidity with neuropsychiatric disorders (e.g., depression, anxiety, motor disturbance, euphoria, and others) [5]. A previous study by the Texas Alzheimer’s Research and Care Consortium (TARCC) reported that Hispanics who showed early signs of cognitive decline tended to have higher rates of depression than their White counterparts [27,28]. In addition, the prevalence of depression in Hispanic females is higher than in Hispanic males [29]. A review article showed an association between chronic discrimination and certain psychiatric conditions, including depression, anxiety and substance use, and physical disorders [30].

Moreover, risk factors associated with demographic factors and genetic influences may increase the susceptibility to developing AD and neuropsychiatric disorders. Among all candidate genes (e.g., APOE, APP, PSEN1/2, GAPDH, TREM2, and ESR genes), the APOE gene showed consistent associations with dementia-related phenotypes and neuropsychiatric diseases (e.g., anxiety, depression), particularly in aging populations [4,31,32,33,34]. A review paper showed that the APOE gene is associated with AD and psychiatric disorders [35]. APOE exhibits three alleles: ε3, ε2, and ε4. The allele known as the neutral allele is APOE-ε3, since it is present in about 75% of the general population [36]. APOE-ε2 is believed to be a protective allele against neurological diseases. Moreover, a meta-analysis study on 20 cohorts from European, East Asian, and African American ancestry demonstrated that those carrying APOE-ε2 had a higher prevalence of surviving the 90th and 99th percentile age [37]. However, this association has not been seen among the Hispanic population, even with their low mortality rates [38]. Both age and the APOE-ε4 allele are known to be risk factors for neurodegenerative diseases [39]. One study reported that at least 60% of AD participants were APOE-ε4 carriers within the White population [40]. The estimated prevalence of APOE-ε4 within Hispanics is approximately 15–20%, but due to admixture patterns, this might not be an accurate representation [12]. In addition, a comprehensive meta-analysis was conducted to investigate the association between the APOE genotype and AD across various ethnic and racial populations, including Hispanic subjects both with and without AD. Among the Hispanic subjects, 19.2% of individuals diagnosed with AD (*N* = 261) carried the APOE-ε4 allele, while 11% of the control subjects (*N* = 267) without AD had the APOE-ε4 allele [41]. The authors concluded that APOE-ε4 was lower in Hispanic AD patients (19.2%) than in White AD patients (36.7%) and more weakly associated with AD as compared to non-Hispanic White [41].

Several association studies of the APOE gene with AD-related phenotypes in Hispanics have been reported, but limited research has been conducted with consistent findings.

APOE-ε4 allele is associated with increased disease risk, however, its distribution is not well-understood among Hispanic population which is highly admixed [38]. Overall, a lack of studies on the associations between the APOE gene with AD-related phenotypes and psychiatric disorders in the Hispanic population was observed [42]. No studies on the APOE locus associated with depression and anxiety in Hispanics were reported based on a PubMed Search on 20 May 2023. Therefore, the objective of this study is to further understand the APOE alleles’ impact on AD-related traits, depression, and anxiety.

## 2. Materials and Methods

This study analyzed data collected by the TARCC (*N* = 1320) in combination with our data, Initial Study of Longevity and Dementia from the Rio Grande Valley (ISLD-RGV, *N* = 62), a total of 1382 subjects. This study’s protocols were approved by the corresponding Institutional Ethics Committees and Institutional Review Boards. A written consent form was obtained from each participant or their legally authorized proxies were gathered before data collection began, as in previous studies [43,44].

The 1st set of data was from ISLD-RGV. Controls were matched to cases based on age, gender, and ethnicity (U.S. Hispanic subjects). The patients with AD, MCI, neuropsychiatric disorders, and healthy subjects were recruited from Brownsville and McAllen, Texas. Participants were recruited from adult day care centers and local health care providers. Questionnaires regarding lifestyle (10 questions [45]) and medical history (modified based on [46]) were collected during the interview. A doctor’s diagnosis was needed to classify if cognitive dysfunctions, e.g., AD, were present; however, neuropsychiatric disorders were self-reported.

The 2nd set of data was collected from the TARCC, an ethnically diverse convenience sample with annual longitudinal follow-up described in detail in [47,48,49]. TARCC is a longitudinally followed convenience sample of elderly persons diagnosed with AD, MCI, or control subjects recruited from five Texas medical schools. Participants underwent a standardized annual examination, including a medical evaluation, neuropsychological testing, and clinical interview. Diagnosis of depression for the TARCC subjects was assessed based on the Geriatric Depression Scale (GDS) [50]. The Neuropsychiatric Inventory Questionnaire (NPI-Q) was used to assess the diagnosis of anxiety (https://sites.cscc.unc.edu/aric/sites/default/files/public/forms/NPI_0.pdf (accessed on 16 January 2023)).

The inclusion criteria for both datasets were individuals with and without diseases (as control) and aged 60 or older belonging to the Hispanic/Latino population. For patients, we focused on the subjects who have cognitive impairment and/or any other neuropsychiatric disorders, including depression or anxiety. The exclusion criteria included individuals younger than 60 and non-Hispanic/Latino subjects.

The diagnoses of AD, MCI, depression, or anxiety for ISLD-RGV were based on self-reports; however, the diagnoses of AD, MCI, depression, or anxiety for the TARCC data were based on the National Institute for Neurological Communicative Disorders and Stroke-Alzheimer’s Disease and Related Disorders Association criteria for AD [51] and site-specific consensus-based clinical diagnoses derived from all available information but without reliance on specific neurocognitive tests and/or cut scores [48,49]. Both databases’ demographic information was based on self-reported age, gender, education, ethnicity, and self-explanatory information.

### 2.1. DNA Isolation and Genotyping

DNA extraction was performed using saliva samples for ISLD-RGV data (*N* = 62 participants) using Oragene DISCOVER (OGR-500). DNA isolation from saliva was performed following the standardized laboratory protocol described by DNA Genotek using PrepIT^®^•L2P. DNA isolation and genotyping of the TARCC’s samples have been described in detail in previous studies [52,53].

The genotypes for the APOE gene were based on the results of Affymetrix Genome-Wide Human single nucleotide polymorphism (SNP) Array 6.0 to collect SNP rs7412 and rs429358 data for TARCC data, as previously reported [54]. There were missing genotype data from ISLD-RGV. APOE alleles (ε3, ε2, and ε4) were determined by SNPs rs7412 and rs429358. SNP data were identified as either absent (ε2−, ε3−, ε4−) or present, being heterozygous or homozygous (ε2+, ε3+, ε4+).

### 2.2. Statistical Analyses

Statistical analysis was performed according to APOE carriers (ε2+, ε3+, ε4+) and non-carriers (ε2−, ε3−, and ε4−). Genotype data were tested for Hardy–Weinberg equilibrium (HWE) before data analysis (p_HWE_ < 0.05). The categorical variables were presented in their raw values along with the proportions, while continuous variables were presented in the form of mean ± standard deviation (SD). Chi-square tests were used to examine the associations of categorical variables with binary outcomes such as AD diagnostics, anxiety, and depression. Independent samples *t*-test was used to determine the differences in continuous variables between two groups.

Multivariable logistic regression models were used to test whether the APOE alleles were independently associated with several phenotypes (e.g., AD, MCI, anxiety, depression, and memory decline), adjusting for all the potential risk factors (e.g., sex, age, and education). The odds ratio (OR) and 95% confidence interval (CI) were used to determine the risk value between alleles and phenotypes. Differences with two-tailed probability values of *p* < 0.05 were accepted as statistically significant. All analyses were performed using The Statistical Package for Social Sciences (SPSS) version 26.

### 2.3. Power Analyses

As in our previous study [55], power analyses were conducted for known genes, as estimated using a case-control study design for discrete traits using the Genetic Power Calculator [56], based on sample size, an average of 1000 control subjects and 300 cases using a variance components analysis with the following parameters: disease prevalence, 0.01; D′0 between disease and SNP alleles, 0.8; α, 0.05; and case-control statistics of allelic 1 df test (B versus b). The power to detect association was estimated as 78.2%, based on the total sample of 1333 subjects, including 300 affected subjects with disease phenotypes (e.g., AD or MCI) and marker allele frequency of 0.2 since these frequencies are the minor allele frequencies of the tested SNPs/markers.

## 3. Results

### 3.1. APOE Allele Distributions and Association with AD and with Anxiety and Depression

**Figure 1:**
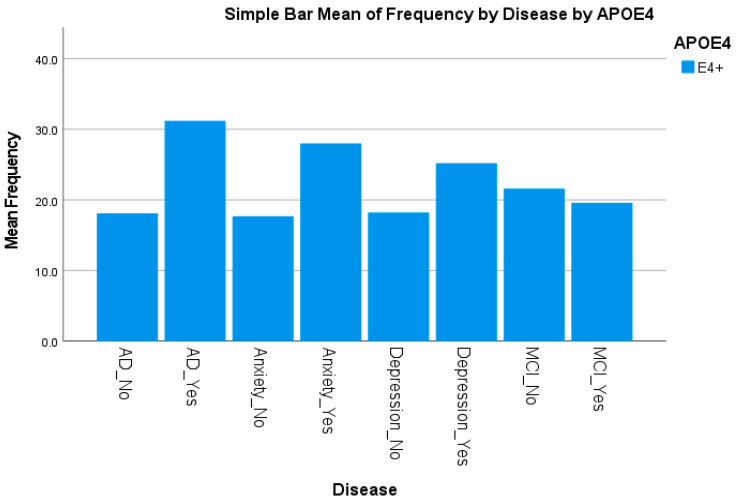
APOE-ε4 frequency by AD status and mental health disorders.

This study included 1382 Hispanic participants collected by the TARCC and ISLD-RGV; from the total participants, only 1320 were genotyped and assigned their corresponding APOE allele status. APOE genotypes were tested with Hardy–Weinberg equilibrium (HWE) (p_HWE_ > 0.05). In total, 921 (69.8%) of the subjects were females, and 399 (30.2%) of the subjects were males, with a mean age of 68.95 ± 9.47 years. The clinical phenotypic frequencies based on APOE-ε4 allele status for the 1,320 participants are displayed in Table 1. A total of 20.6% of females and 22.2% of males carried at least one APOE-ε4 allele (Table 1). The APOE-ε4 allele was not only associated with AD (31.2%) compared with normal cognition (18.1%) (*p* < 0.0001) but was also associated with depression (*p* = 0.002) and anxiety (*p* < 0.0001). Figure 1 shows the APOE-ε4 frequency by AD status and mental health disorders (e.g., depression or anxiety). No difference in APOE-ε4 status was observed for BMI, MCI, and education.

The APOE-ε3 allele maintained the highest prevalence, with at least 90% of the participants being APOE-ε3 carriers. In addition, the APOE allele frequencies of the 1019 Hispanic control subjects were assessed, with APOE-ε3 (89.5%) showing the highest frequency, followed by APOE-ε4 (18.1%) and APOE-ε2 (6.8%).

### 3.2. APOE and Demographic and Health Characteristics with AD Diagnosis

The demographic characteristics of the AD, MCI, and the control groups are shown in Table 2. Although females were the predominant sex within the AD group, males (*N* = 105, 26.3%) were more likely to suffer from AD compared to females (*N* = 196, 21.3%) (*p* = 0.045). The cognitive statuses of the 1,320 participants were classified as 301 AD (22.8%) and 327 MCI (24.8%). The mean age of AD cases was 77.54 ± 7.73 years and was statistically significantly older than the control subjects (67.83 ± 9.26, *p* < 0.0001). The mean age of the MCI cases was 73.97 ± 8.67, 6 years older than the controls, but compared to the AD cases, they were approximately 4 years younger. The prevalence of AD in depression (34.0%) and anxiety (40.3%) was significant higher as compared to the control group (*p* < 0.0001). The same trends were observed in the MCI group (*p* = 0.023 and *p* = 0.004, respectively)

### 3.3. APOE and Demographic Characteristics with Two Neuropsychiatric Disorders

The frequencies of the APOE alleles and demographic characteristics within neuropsychiatric disorders are shown in Table 3. The independent *t*-test showed that age was associated with anxiety (*p* < 0.0001) and depression (*p* < 0.0001). Furthermore, the APOE-ε4 allele showed significant associations with anxiety (*p* < 0.0001) and depression (*p* = 0.002). APOE-ε3 was shown to be significantly associated with depression (*p* = 0.002). Finally, highly educated Hispanics were less likely to suffer from depression (*p* = 0.04).

### 3.4. Logistic Regression Analysis of AD diagnosis

After adjusting for covariates, multivariable logistic regression analysis further supported the association between APOE-ε4 and AD (OR = 1.54, *p* = 0.033, Table 4), whereas no association was found between MCI and APOE-ε4 (*p* = 0.156, Table 4). Furthermore, age (OR = 1.16, *p* < 0.0001), anxiety (OR = 2.01, *p* < 0.0001), and depression (OR = 1.71, *p* = 0.004) were associated with AD. However, anxiety (OR = 1.24, *p* = 0.196) and depression (OR = 1.12, *p* = 0.455) did not maintain significant associations with MC in the multivariable logistic regression analyses.

### 3.5. Logistical Regression Analysis of Neuropsychiatric Disorders

Table 5 presents the results of multivariable logistic regression analyses of depression and anxiety. After adjusting for covariates, the APOE-ε4 remained statistically significantly associated with anxiety (OR = 1.71, *p* = 0.0002) and depression (OR = 1.48, *p* = 0.004). Furthermore, age was associated with anxiety (*p* < 0.0001) and depression (*p* < 0.0001).

## 4. Discussion

The major findings of this study include that (1) APOE-ε4 is associated with AD in the elderly Hispanic participants, which correlates with previous studies, including a meta-analysis study that showed Whites, African Americans, and Asians who were APOE-ε4 carriers had a high risk for AD [41,57]. Moreover, a study on Caribbean Hispanics indicated that APOE-ε4 carriers were at risk for AD due to their genetic diversity background, local ancestry, or haplotypic background [58]. (2) The APOE-ε4 allele is associated with anxiety and depression in the current studied population, Hispanics, which is the first positive association reported in this population, although APOE-ε4-associated anxiety and depression was reported in non-Hispanic populations [59,60]. This study provides the future possibility of further research on the APOE allele status as a genetic marker for early diagnosis of dementia-related problems, anxiety, and/or depression.

The APOE allele frequencies of the Hispanic participants showed that 89.5% carried APOE-ε3, followed by 18.1% for APOE-ε4, and 6.8% for APOE-ε2, similar to previous studies in Hispanics [38,58]. The frequency of the APOE-ε4 allele (18.1%) was slightly lower in Hispanics as compared to other populations, including in the National Cell Repository for Alzheimer’s Disease Family study (NIALOAD) on Europeans (32%). As predicted, APOE-ε4 was more frequent in patients with AD compared to the control subjects in our studied Hispanic population, which shows a similar finding to a previous study [61], Caribbean Hispanics [58], and Caribbean Hispanics and Hispanic Americans [62]. Importantly, Hispanic patients with anxiety, depression, and MD were more likely to be APOE-ε4 carriers than non-APOE-ε4 carriers. However, a future study for confirmation is necessary. Although we did not observe APOE-ε2 as a protective allele for AD or MCI, previous studies suggest that APOE-ε2 may have protective effects against AD in Hispanics [38,61] and Caribbean Hispanics [58]. However, APOE-ε2′s effect on cognition, dementia-related traits, and/or other AD imaging markers (e.g., brain structure, function, and metabolism) were inconsistent and, therefore, inconclusive [63]. Furthermore, APOE-ε3 revealed its significance relating to depression after controlling potential confounding factors in our current study. Previous reports focused on the association of APOE alleles (ε2, ε3, ε4) and AD or other traits in the Hispanic population have found inconsistent results, partly due to the high heterogeneity in Hispanic populations (e.g., admixture, diet, environment, vascular risk factors) [63]. Nevertheless, further studies with a large sample are required for confirmation. Hispanics with slightly higher levels of education were found to be less prone to experiencing depression in our current study.

In terms of the association of the APOE gene with neuropsychiatric disorders, a meta-analysis showed an association between the APOE-ε3/ε4 genotype and depression in White and Asian populations based on 20 studies [64]. In our current study, AD patients were associated with higher frequencies of anxiety and depression. Patients with AD (22.8%) and comorbid with depression (34.0%) and anxiety (40.3%) are shown in Table 2. As previously indicated, more than half of the AD cases showed symptoms of depression and anxiety, which were less frequent in MCI cases compared with AD cases. The same trends were observed among the patients with MCI (24.8%) with comorbidity with depression (28%) and anxiety (29.6%) in the MCI group.

However, a recent meta-analysis study indicated that there is no evidence that APOE-ε4 allele carriership is associated with depression and/or anxiety in non-Hispanic populations [65]. A study suggested that individuals with depression have a higher prevalence of developing AD, concluding that depression found later in life may be a risk factor for developing AD in mixed populations [66]. However, relationships between depression and AD are difficult to interpret due to the similarity of their clinical presentations [25]. Although there were inconsistent findings, a recent study based on 15,640 individuals demonstrated a significant genomic overlap with genetic variants (including APOE gene) associated with depression and cognitive functioning in unknown populations [67]. Our current study is the first study which examines the association between the APOE-ε4 allele and depression and anxiety in the Hispanic population. However, we are aware of phenotype heterogeneity among the U.S Hispanic population. Relationships between certain psychiatric disorders (e.g., anxiety, depression) and AD may become clearer if studied in preclinical AD participants or in participants without AD. Further studies with a large sample size are required to confirm the association of the APOE-ε4 allele with these neuropsychiatric disorders in the Hispanic population.

We are aware of the limitations of our current study. (1) Although we observed the comorbidity of AD and/or MCI with depression and/or anxiety, which has been widely reported [68], we did not test the causal relationship between these psychiatric conditions and AD. Alternatively, their co-occurrence might be due to confounding or common risk factors such as aging [69]. In addition, confounding factors for AD, MCI, or neuropsychiatric disorders also include alcohol, alcoholism, social isolation, and/or lifestyle. (2) The sample size in our current study is small (*N* = 1382), which may be limiting for genetic studies, potentially resulting in false-negative or positive findings. Thus, a future study on U.S. Hispanics with a larger sample size is needed to confirm our current findings. There was 78% statistical power; however, it is known that the U.S Hispanic population is heterogeneous geographically, culturally, racially, and genetically [70]. It is plausible to consider that the expression of APOE genetic susceptibility may vary inconsistently, potentially influenced by sub-Hispanic populations or specific population origins present in the two data collection sites of our study. (3) In terms of the association between the APOE-ε4 allele and depression and anxiety, the occurrence of mental disorders is high across AD and MCI. In our current study, participants with depression and/or anxiety are phenotypically heterogenous and comorbid with AD and MCI. For example, among our studied population, 432 cases were reported with anxiety (28%), 174 with AD (58%), 128 with MCI (39%), and 130 control subjects without AD were reported (25%). It is unclear if the APOE-ε4 allele is associated with these psychiatric conditions, AD, or comorbidity of both. Thus, a future association study between APOE alleles and these neuropsychiatric traits without AD in the Hispanic population is needed. (4) Phenotypic and genotypic heterogeneity (certain variables, e.g., age, sex, and level of education and sociability) may exist between the two datasets. Future studies which specifically focus on investigating the association between APOE and diseases among different Hispanic origins and sub-populations are needed.

## 5. Conclusions

We present compelling evidence indicating that APOE-ε4 significantly raises the risk of developing AD among individuals of Hispanic descent. Furthermore, our study reveals, for the first time, that APOE alleles are associated with elevated risks of anxiety and depression in the Hispanic population. These novel findings call for further research to validate and expand upon the current observations. However, further studies are needed to confirm our current findings regarding this population.

## Figures and Tables

**Table 1:** APOE-ε4 allele status for demographic and health characteristics.

	APOE-ε4−	APOE-ε4+	*p* Value *
Age (mean ± SD)	68.96 ± 9.46	70.80 ± 9.48	0.004
Females (*N*, %) Males (*N*, %)	731 (79.4%) 311 (77.9%)	190 (20.6%) 88 (22.2%)	0.560
Education (mean ± SD)	10.17 ± 4.61	10.41 ± 4.74	0.439
BMI (mean ± SD)	31.92 ± 6.56	31.03 ± 6.32	0.042
AD (*N*, %) Control (*N*, %)	207 (68.8%) 835 (81.1%)	94 (31.2%) 184 (18,1%)	<0.0001
MCI (*N*, %) Control (*N*, %)	263 (80.4%) 779 (78.4%)	64 (19.6%) 214 (21.6%)	0.447
Anxiety (*N*, %) Control (*N*, %)	311 (72.0%) 731 (82.3%)	121 (28.0%) 157 (17.7%)	<0.0001
Depression (*N*, %) Control (*N*, %)	400 (74.8%) 642 (81.7%)	135 (25.2%) 143 (18.2%)	0.002

* *p* value, *t* test for continuous variables, and χ^2^ test for categorical variables. Carrying at least one copy of APOE-ε4 (APOE-ε4+). The APOE-ε4 is absent (APOE-ε4−). AD, Alzheimer’s disease; MCI, mild cognitive impairment; BMI, body mass index. Numerical values are expressed as the mean ± SD.

**Table 2:** APOE and demographic and health characteristics with AD diagnosis.

	Controls	AD Cases	*p* *	Controls	MCI Cases	*p* *
Age (mean ± SD)	66.93 ± 8.58	77.53 ± 7.73	<0.0001	67.83 ± 9.26	73.97 ± 8.67	<0.0001
Male (*N*, %) Female (*N*, %)	294 (73.7%) 725 (78.7%)	105 (26.3%) 196 (21.3%)	0.045	289 (72.4%) 704 (76.4%)	110 (27.6%) 217 (23.6%)	0.121
Education (mean ± SD)	10.19 ± 4.70	10.35 ± 4.44	0.599	10.22 ± 4.59	10.21 ± 4.80	0.983
Anxiety (*N*, %) Control (*N*, %)	258 (59.7%) 761(85.7%)	174 (40.3%) 127 (14.3%)	<0.0001	304 (70.4%) 689 (77.6%)	128 (29.6%) 199 (22.4%)	0.004
Depression (*N*, %) Control (*N*, %)	353 (66.0%) 666 (84.8%)	182 (34.0%) 119 (15.2%)	<0.0001	385 (72.0%) 608 (77.5%)	150 (28.0%) 177 (22.5%)	0.023
APOE-ε3+ (*N*, %) APOE-ε3− (*N*, %)	912 (76.4%) 107 (84.3%)	281 (23.6%) 20 (15.7%)	0.046	892 (74.8%) 101(79.5%)	301 (25.2%) 26 (20.5%)	0.579
APOE-ε4+ (*N*, %) APOE-ε4− (*N*, %)	184 (66.2%) 835 (80.1%)	94 (33.8%) 207 (19.9%)	<0.0001	214 (77.0%) 779 (74.8%)	64 (23.0%) 263 (25.2%)	0.447

* *p* value, *t*-test for continuous variables, and χ^2^ test for categorical variables. AD, Alzheimer’s disease; MCI, mild cognitive impairment; *N*, number of patients; %, percentage; numerical values are expressed as the mean ± SD.

**Table 3:** APOE and demographic characteristics with neuropsychiatric disorders.

	Controls	Anxiety	*p* *	Controls	Depression	*p* *
Age (mean ± SD)	68.09 ± 9.37	71.93 ± 9.22	<0.0001	68.37 ± 9.57	70.78 ± 9.20	<0.0001
Male (*N*, %) Female (*N*, %)	274 (68.7%) 614 (66.7%)	125 (31.3%) 307 (33.3%)	0.476	246 (61.7%) 539 (58.5%)	153 (38.3%) 382 (41.5%)	0.287
BMI (mean ± SD)	31.89 ± 6.51	31.42 ± 6.54	0.227	31.47 ± 6.43	32.13 ± 6.63	0.071
Education (mean ± SD)	10.25 ± 4.69	10.16 ± 4.53	0.734	10.44 ± 4.73	9.90 ± 4.48	0.040
APOE-ε3+ (*N*, %) APOE-ε3− (*N*, %)	795 (66.6%) 93(73.2%)	398 (33.4%) 34 (26.8%)	0.132	693 (58.1%) 92 (72.4%)	500 (41.9%) 35 (27.6%)	0.002
APOE-ε4+ (*N*, %) APOE-ε4− (*N*, %)	157 (56.5%) 731 (70.2%)	121 (43.5%) 311 (29.8%)	<0.0001	143 (51.4%) 642 (61.6%)	135 48.6%) 400 (38.4%)	0.002

* *p* value, *t*-test for continuous variables, and χ^2^ test for categorical variables. BMI, body mass index. Numerical values are expressed as the mean ± SD.

**Table 4:** Logistic regression analysis of AD diagnosis.

	AD vs. Control		MCI vs. Control	
	*OR* (95% *CI*)	*p*	*OR* (95% *CI*)	*p*
Age	1.16 (1.13, 1.18)	<0.0001	1.08 (1.06, 1.09)	<0.0001
Sex (ref = female)	1.20 (0.80, 1.58)	0.515	1.13 (0.85, 1.51)	0.400
BMI	0.99 (0.96, 1.02)	0.399	1.03 (1.01, 1.05)	0.015
Education	1.02 (0.98, 1.05)	0.399	1.00 (0.97, 1.03)	0.989
Anxiety (ref = no)	2.01 (1.39, 2.91)	<0.0001	1.24 (0.90, 1.72)	0.196
Depression (ref = no)	1.71 (1.19, 2.47)	0.004	1.12 (0.83, 1.53)	0.455
APOE-ε4+ (ref = APOE-ε4−)	1.50 (1.03, 2.18)	0.033	0.79 (0.56, 1.10)	0.156

*t*-test for continuous variables. AD, Alzheimer’s; MCI, mild cognitive impairment; disease *B* unstandardized regression weight; *OR*, odds ratio; *CI*, confidence interval. BMI, body mass index. Carrying at least one copy of APOE-ε4 (APOE-ε4+).

**Table 5:** Logistical regression analysis of neuropsychiatric disorders.

	Anxiety vs. Control		Depression vs. Control	
	*OR* (95% *CI*)	*p*	*OR* (95% *CI*)	*p*
Age	1.05 (1.03, 1.06)	<0.0001	1.03 (1.02, 1.04)	<0.0001
Education	1.00 (0.97, 1.02)	0.723	0.98 (0.95, 1.00)	0.054
Sex (ref = female)	0.82 (0.63, 1.06)	0.130	0.86 (0.67, 1.10)	0.226
BMI	1.00 (0.98, 1.02)	0.918	1.02 (1.01, 1.04)	0.009
APOE-ε4+ (ref = APOE-ε4−)	1.71 (1.29, 2.26)	<0.0001	1.48 (1.13, 1.95)	0.004

*t*-test for continuous variables. *B* unstandardized regression weight; *OR*, odds ratio; *CI*, confidence interval. BMI, body mass index. Carrying at least one copy of APOE-ε4 (APOE-ε4+).

## Data Availability

Part of the data was from the Texas Alzheimer’s Research and Care Consortium (TARCC) at https://www.txalzresearch.org/ accessed on 6 January 2023.

## References

[1] International A.D., Wimo A., Ali G.-C., Guerchet M., Prince M., Prina M., Wu Y.-T. (2015). World Alzheimer Report 2015: The Global Impact of Dementia: An Analysis of Prevalence, Incidence, Cost and Trends.

[2] Gimson A., Schlosser M., Huntley J.D., Marchant N.L. (2018). Support for Midlife Anxiety Diagnosis as an Independent Risk Factor for Dementia: A Systematic Review. BMJ Open.

[3] Peters R., Booth A., Rockwood K., Peters J., D’Este C., Anstey K.J. (2019). Combining Modifiable Risk Factors and Risk of Dementia: A Systematic Review and Meta-Analysis. BMJ Open.

[4] Ferretti L., McCurry S.M., Logsdon R., Gibbons L., Teri L. (2001). Anxiety and Alzheimer’s Disease. J. Geriatr. Psychiatry Neurol..

[5] Zhao Q.-F., Tan L., Wang H.-F., Jiang T., Tan M.-S., Tan L., Xu W., Li J.-Q., Wang J., Lai T.-J. (2016). The Prevalence of Neuropsychiatric Symptoms in Alzheimer’s Disease: Systematic Review and Meta-Analysis. J. Affect. Disord..

[6] National Institute on Aging What Is Mild Cognitive Impairment?. http://www.nia.nih.gov/health/what-mild-cognitive-impairment.

[7] Langa K.M., Levine D.A. (2014). The Diagnosis and Management of Mild Cognitive Impairment: A Clinical Review. J. Am. Med. Assoc..

[8] Plassmann H., O’Doherty J., Shiv B., Rangel A. (2008). Marketing Actions Can Modulate Neural Representations of Experienced Pleasantness. Proc. Natl. Acad. Sci. USA.

[9] Gupta S. (2021). Racial and Ethnic Disparities in Subjective Cognitive Decline: A Closer Look, United States, 2015–2018. BMC Public Health.

[10] Eshkoor S.A., Hamid T.A., Mun C.Y., Ng C.K. (2015). Mild Cognitive Impairment and Its Management in Older People. Clin. Interv. Aging.

[11] Mild Cognitive Impairment More Common in Older Men than Older Women. https://www.nih.gov/news-events/news-releases/mild-cognitive-impairment-more-common-older-men-older-women.

[12] González H.M., Tarraf W., Schneiderman N., Fornage M., Vásquez P.M., Zeng D., Youngblood M., Gallo L.C., Daviglus M.L., Lipton R.B. (2019). Prevalence and Correlates of Mild Cognitive Impairment among Diverse Hispanics/Latinos: Study of Latinos-Investigation of Neurocognitive Aging Results. Alzheimer’s Dement..

[13] Salazar R., Dwivedi A.K., Royall D.R. (2016). Cross-Ethnic Differences in the Severity of Neuropsychiatric Symptoms in Persons With Mild Cognitive Impairment and Alzheimer’s Disease. J. Neuropsychiatry Clin. Neurosci..

[14] Centers for Disease Control and Prevention U.S. Burden of Alzheimer’s Disease, Related Dementias to Double by 2060 | CDC Online Newsroom | CDC. https://www.cdc.gov/media/releases/2018/p0920-alzheimers-burden-double-2060.html.

[15] Lyketsos C.G., Carrillo M.C., Ryan J.M., Khachaturian A.S., Trzepacz P., Amatniek J., Cedarbaum J., Brashear R., Miller D.S. (2011). Neuropsychiatric Symptoms in Alzheimer’s Disease. Alzheimer’s Dement..

[16] Ng K.P., Pascoal T.A., Mathotaarachchi S., Chan Y.H., Jiang L., Therriault J., Benedet A.L., Shin M., Kandiah N., Greenwood C.M.T. (2021). Neuropsychiatric Symptoms Are Early Indicators of an Upcoming Metabolic Decline in Alzheimer’s Disease. Transl. Neurodegener..

[17] Gil M., Alliey-Rodriguez N., Lopez-Alvarenga J.C., Diego V., Gaona C.A., Mata L., Pirela R.V., Chavez C.A., de Erausquin G.A., Melgarejo J.D. (2021). Neuropsychiatric Symptoms Among Hispanics: Results of the Maracaibo Aging Study. J. Alzheimer’s Dis..

[18] Alegría M., Mulvaney-Day N., Torres M., Polo A., Cao Z., Canino G. (2007). Prevalence of Psychiatric Disorders across Latino Subgroups in the United States. Am. J. Public Health.

[19] Cohen S.D., Cukor D., Kimmel P.L. (2016). Anxiety in Patients Treated with Hemodialysis. Clin. J. Am. Soc. Nephrol..

[20] The National Institute of Mental Health NIMH Any Anxiety Disorder. https://www.nimh.nih.gov/health/statistics/any-anxiety-disorder.shtml.

[21] Bandelow B., Michaelis S. (2015). Epidemiology of Anxiety Disorders in the 21st Century. Dialogues Clin. Neurosci..

[22] Baxter A.J., Scott K.M., Vos T., Whiteford H.A. (2013). Global Prevalence of Anxiety Disorders: A Systematic Review and Meta-Regression. Psychol. Med..

[23] Anxiety Disorder Statistics 2020—U.S. And Worldwide. https://mindfulsearching.com/anxiety-statistics/.

[24] Vlainić J., Šuran J., Vlainić T., Vukorep A.L. (2016). Probiotics as an Adjuvant Therapy in Major Depressive Disorder. Curr. Neuropharmacol..

[25] Leyhe T., Reynolds C.F., Melcher T., Linnemann C., Klöppel S., Blennow K., Zetterberg H., Dubois B., Lista S., Hampel H. (2017). A Common Challenge in Older Adults: Classification, Overlap, and Therapy of Depression and Dementia. Alzheimer’s Dement..

[26] Avenevoli S., Swendsen J., He J.-P., Burstein M., Merikangas K. (2015). Major Depression in the National Comorbidity Survey-Adolescent Supplement: Prevalence, Correlates, and Treatment. J. Am. Acad. Child Adolesc. Psychiatry.

[27] O’Bryant S.E., Johnson L., Balldin V., Edwards M., Barber R., Williams B., Devous M., Cushings B., Knebl J., Hall J. (2013). Characterization of Mexican Americans with Mild Cognitive Impairment and Alzheimer’s Disease. J. Alzheimer’s Dis..

[28] Camacho A., Tarraf W., Jimenez D.E., Gallo L.C., Gonzalez P., Kaplan R.C., Lamar M., Khambaty T., Thyagarajan B., Perreira K.M. (2018). Anxious Depression and Neurocognition among Middle-Aged and Older Hispanic/Latino Adults: Hispanic Community Health Study/Study of Latinos (HCHS/SOL) Results. Am. J. Geriatr. Psychiatry.

[29] Vega W., Kolody B., Aguilar-Gaxiola S., Alderete E., Catalano R., Caraveo-Anduaga J. (1998). Lifetime Prevalence of DSM-III-R Psychiatric Disorders Among Urban and Rural Mexican Americans in California. Arch. Gen. Psychiatry.

[30] Brenes F. (2019). Hispanics, Mental Health, and Discriminating Policies: Brief Report. Hisp. Health Care Int..

[31] Armstrong R.A. (2019). Risk Factors for Alzheimer’s Disease. Folia Neuropathol..

[32] Buchman A.S., Bennett D.A. (2011). Loss of Motor Function in Preclinical Alzheimer’s Disease. Expert Rev. Neurother..

[33] Gottschalk W.K., Mihovilovic M., Roses A.D., Chiba-Falek O. (2016). The Role of Upregulated APOE in Alzheimer’s Disease Etiology. J. Alzheimer’s Dis. Park..

[34] Tsang R.S.M., Mather K.A., Sachdev P.S., Reppermund S. (2017). Systematic Review and Meta-Analysis of Genetic Studies of Late-Life Depression. Neurosci. Biobehav. Rev..

[35] Forero D.A., López-León S., González-Giraldo Y., Dries D.R., Pereira-Morales A.J., Jiménez K.M., Franco-Restrepo J.E. (2018). APOE Gene and Neuropsychiatric Disorders and Endophenotypes: A Comprehensive Review. Am. J. Med. Genet. Part B Neuropsychiatr. Genet..

[36] Wu L., Zhang X., Zhao L. (2018). Human ApoE Isoforms Differentially Modulate Brain Glucose and Ketone Body Metabolism: Implications for Alzheimer’s Disease Risk Reduction and Early Intervention. J. Neurosci..

[37] Deelen J., Evans D.S., Arking D.E., Tesi N., Nygaard M., Liu X., Wojczynski M.K., Biggs M.L., van der Spek A., Atzmon G. (2019). A Meta-Analysis of Genome-Wide Association Studies Identifies Multiple Longevity Genes. Nat. Commun..

[38] González H.M., Tarraf W., Jian X., Vásquez P.M., Kaplan R., Thyagarajan B., Daviglus M., Lamar M., Gallo L.C., Zeng D. (2018). Apolipoprotein E Genotypes among Diverse Middle-Aged and Older Latinos: Study of Latinos-Investigation of Neurocognitive Aging Results (HCHS/SOL). Sci. Rep..

[39] Yin Y., Wang Z., Wang Z. (2018). ApoE and Neurodegenerative Diseases in Aging. Aging and Aging-Related Diseases: Mechanisms and Interventions.

[40] Muñoz S.S., Garner B., Ooi L. (2019). Understanding the Role of ApoE Fragments in Alzheimer’s Disease. Neurochem. Res..

[41] Farrer L.A., Cupples L.A., Haines J.L., Hyman B., Kukull W.A., Mayeux R., Myers R.H., Pericak-Vance M.A., Risch N., van Duijn C.M. (1997). Effects of Age, Sex, and Ethnicity on the Association Between Apolipoprotein E Genotype and Alzheimer Disease: A Meta-Analysis. J. Am. Med. Assoc..

[42] Campos M., Edland S.D., Peavy G.M. (2013). An Exploratory Study of APOE-Ε4 Genotype and Risk of Alzheimer’s Disease in Mexican Hispanics. J. Am. Geriatr. Soc..

[43] Grady C. (2015). Institutional Review Boards. Chest.

[44] McMillan G. (2020). IRB Policies for Obtaining Informed Consent from Non-English-Speaking People. Ethics Hum. Res..

[45] Godwin M., Streight S., Dyachuk E., van den Hooven E.C., Ploemacher J., Seguin R., Cuthbertson S. (2008). Testing the Simple Lifestyle Indicator Questionnaire: Initial Psychometric Study. Can. Fam. Physician.

[46] Steidel A.G.L., Contreras J.M. (2003). A New Familism Scale for Use with Latino Populations. Hisp. J. Behav. Sci..

[47] O’Bryant S.E., Waring S.C., Cullum C.M., Hall J., Lacritz L., Massman P.J., Lupo P.J., Reisch J.S., Doody R., Texas Alzheimer’s Research Consortium (2008). Staging Dementia Using Clinical Dementia Rating Scale Sum of Boxes Scores: A Texas Alzheimer’s Research Consortium Study. Arch. Neurol..

[48] O’Bryant S.E., Xiao G., Barber R., Reisch J., Doody R., Fairchild T., Adams P., Waring S., Diaz-Arrastia R., Texas Alzheimer’s Research Consortium (2010). A Serum Protein-Based Algorithm for the Detection of Alzheimer Disease. Arch. Neurol..

[49] Royall D.R., Bishnoi R.J., Palmer R.F., Alzheimer’s Disease Neuroimaging Initiative (2019). Blood-Based Protein Predictors of Dementia Severity as Measured by δ: Replication across Biofluids and Cohorts. Alzheimer’s Dement..

[50] Maixner S.M., Burke W.J., Roccaforte W.H., Wengel S.P., Potter J.F. (1995). A Comparison of Two Depression Scales in a Geriatric Assessment Clinic. Am. J. Geriatr. Psychiatry.

[51] McKhann G., Drachman D., Folstein M., Katzman R., Price D., Stadlan E.M. (1984). Clinical Diagnosis of Alzheimer’s Disease: Report of the NINCDS-ADRDA Work Group under the Auspices of Department of Health and Human Services Task Force on Alzheimer’s Disease. Neurology.

[52] Hall J.R., Vo H.T., Johnson L.A., Wiechmann A., O’Bryant S.E. (2012). Boston Naming Test: Gender Differences in Older Adults with and without Alzheimer’s Dementia. Psychology.

[53] Royall D.R., Palmer R.F. (2019). Selection for Depression-Specific Dementia Cases with Replication in Two Cohorts. PLoS ONE.

[54] Research T.A., Consortium C. (2016). Data and Sample Information and Requests.

[55] Xu C., Li P.P., Cooke R.G., Parikh S.V., Wang K., Kennedy J.L., Warsh J.J. (2009). TRPM2 Variants and Bipolar Disorder Risk: Confirmation in a Family-Based Association Study. Bipolar Disord..

[56] Purcell S., Cherny S.S., Sham P.C. (2003). Genetic Power Calculator: Design of Linkage and Association Genetic Mapping Studies of Complex Traits. Bioinformatics.

[57] Turney I.C., Chesebro A.G., Rentería M.A., Lao P.J., Beato J.M., Schupf N., Mayeux R., Manly J.J., Brickman A.M. (2020). APOE Ε4 and Resting-State Functional Connectivity in Racially/Ethnically Diverse Older Adults. Alzheimer’s Dement..

[58] Blue E.E., Horimoto A.R.V.R., Mukherjee S., Wijsman E.M., Thornton T.A. (2019). Local Ancestry at APOE Modifies Alzheimer’s Disease Risk in Caribbean Hispanics. Alzheimer’s Dement..

[59] Lanctôt K.L., Amatniek J., Ancoli-Israel S., Arnold S.E., Ballard C., Cohen-Mansfield J., Ismail Z., Lyketsos C., Miller D.S., Musiek E. (2017). Neuropsychiatric Signs and Symptoms of Alzheimer’s Disease: New Treatment Paradigms. Alzheimer’s Dement..

[60] Teri L., Ferretti L.E., Gibbons L.E., Logsdon R.G., McCurry S.M., Kukull W.A., McCormick W.C., Bowen J.D., Larson E.B. (1999). Anxiety of Alzheimer’s Disease: Prevalence, and Comorbidity. J. Gerontol. Ser. A Biol. Sci. Med. Sci..

[61] Granot-Hershkovitz E., Tarraf W., Kurniansyah N., Daviglus M., Isasi C.R., Kaplan R., Lamar M., Perreira K.M., Wassertheil-Smoller S., Stickel A. (2021). *APOE* Alleles’ Association with Cognitive Function Differs across Hispanic/Latino Groups and Genetic Ancestry in the Study of Latinos-investigation of Neurocognitive Aging (HCHS/SOL). Alzheimer’s Dement..

[62] Llibre-Guerra J.J., Li J., Qian Y., Llibre-Rodriguez J.D.J., Jiménez-Velázquez I.Z., Acosta D., Salas A., Llibre-Guerra J.C., Valvuerdi A., Harrati A. (2023). Apolipoprotein E (*APOE*) Genotype, Dementia, and Memory Performance among Caribbean Hispanic versus US Populations. Alzheimer’s Dement..

[63] Kim H., Devanand D.P., Carlson S., Goldberg T.E. (2022). Apolipoprotein E Genotype E2: Neuroprotection and Its Limits. Front. Aging Neurosci..

[64] Feng F., Lu S.-S., Hu C.-Y., Gong F.-F., Qian Z.-Z., Yang H.-Y., Wu Y.-L., Zhao Y.-Y., Bi P., Sun Y.-H. (2015). Association between Apolipoprotein E Gene Polymorphism and Depression. J. Clin. Neurosci..

[65] Banning L.C.P., Ramakers I.H.G.B., Deckers K., Verhey F.R.J., Aalten P. (2019). Apolipoprotein E and Affective Symptoms in Mild Cognitive Impairment and Alzheimer’s Disease Dementia: A Systematic Review and Meta-Analysis. Neurosci. Biobehav. Rev..

[66] Ownby R.L., Crocco E., Acevedo A., John V., Loewenstein D. (2006). Depression and Risk for Alzheimer Disease. Arch. Gen. Psychiatry.

[67] Brouwer R.M., Klein M., Grasby K.L., Schnack H.G., Jahanshad N., Teeuw J., Thomopoulos S.I., Sprooten E., Franz C.E., Gogtay N. (2022). Genetic Variants Associated with Longitudinal Changes in Brain Structure across the Lifespan. Nat. Neurosci..

[68] Haaksma M.L., Vilela L.R., Marengoni A., Calderón-Larrañaga A., Leoutsakos J.-M.S., Olde Rikkert M.G.M., Melis R.J.F. (2017). Comorbidity and Progression of Late Onset Alzheimer’s Disease: A Systematic Review. PLoS ONE.

[69] Huang J., Zuber V., Matthews P.M., Elliott P., Tzoulaki J., Dehghan A. (2020). Sleep, Major Depressive Disorder, and Alzheimer Disease: A Mendelian Randomization Study. Neurology.

[70] Mehta K.M., Yeo G.W. (2017). Systematic Review of Dementia Prevalence and Incidence in United States Race/Ethnic Populations. Alzheimer’s Dement..

